# Dutch Workflow for Diagnosis and Treatment of Velopharyngeal Insufficiency in Patients with Cleft Palate—A Survey Study

**DOI:** 10.1177/10556656251341757

**Published:** 2025-05-19

**Authors:** JJ Peters, SA van der Kooij, CDL van Gogh, MJ Coerts, JPW Don Griot, CM Moues-Vink, T Wagner, RM Schols, B van Nimmen, MM Pleumeekers, M Ruettermann, EC Paes, TR De Jong, CC Breugem

**Affiliations:** 1 Department of Plastic, Reconstructive and Hand surgery, 26066 Amsterdam UMC location University of Amsterdam, Amsterdam, the Netherlands; 2 Amsterdam Reproduction and Development research institute, Amsterdam, the Netherlands; 3 Department of Ear, Nose and Throat surgery, 1209 Amsterdam UMC location University of Amsterdam, Amsterdam, the Netherlands; 4 Department of Plastic, Reconstructive and Hand surgery, 4480 Leeuwarden Medical Center, Leeuwarden, the Netherlands; 5 Department of Plastic and Reconstructive surgery, 6034 Radboud University Medical Center, Nijmegen, the Netherlands; 6 Department of Plastic, Reconstructive and Hand surgery, 199236 Maastricht University Medical Center, Maastricht, the Netherlands; 7 Department of Plastic, Reconstructive and Hand surgery, 7898 Elisabeth-TweeSteden Ziekenhuis (ETZ), Tilburg, the Netherlands; 8 Department of Plastic, Reconstructive and Hand surgery, 6993 Erasmus Medical Center, Rotterdam, the Netherlands; 9 Department of Plastic Surgery, University of Groningen, 10173 University Medical Center Groningen, Groningen, the Netherlands; 10 Department of Plastic, Reconstructive and Hand surgery, 8124 University Medical Center Utrecht, Utrecht, the Netherlands; 11 Department of Plastic, Reconstructive and Hand surgery, 8772 Isala Hospital, Zwolle, the Netherlands

**Keywords:** cleft palate, cleft lip and palate, velopharyngeal dysfunction

## Abstract

**Objective:**

The aim of this study was to investigate the workflow of cleft teams in the Netherlands with regard to the diagnosis and treatment of velopharyngeal insufficiency (VPI) in patients with cleft palate (CP).

**Design:**

This is a cross-sectional survey study.

**Setting:**

Multicenter study, tertiary hospital setting.

**Participants:**

Ear-nose-throat surgeons, plastic surgeons and speech language pathologists of the eight cleft teams in the Netherlands.

**Interventions:**

A cross-sectional online survey was sent to the participants.

**Main Outcome Measure(s):**

The survey questions covered the following topics: diagnostic tests used to assess VPI, use of classification systems and cut-off values to determine the most optimal treatment, treatment of VPI, and postoperative follow-up.

**Results:**

The response rate was 88% (n = 7 cleft teams). Frequently described diagnostic tests to assess VPI include perceptual speech assessment, mirror test, nasendoscopy, oral inspection, patient-reported outcome measures, nasometry, and videofluoroscopy. Most centers reported that they did not use a classification system to determine the severity of VPI. None of the centers reported to use cut-off values based on the diagnostic tests to determine optimal treatment. The reported minimum duration of speech therapy prior to surgery varied. Many different surgical techniques were reported for the treatment of VPI. Regarding postoperative follow-up, survey responses indicate agreement on the multidisciplinary approach and diagnostic tests used. The timing of the visits varied.

**Conclusion:**

Further standardization of the diagnostic process and treatment workflow of VPI in patients with CP between Dutch cleft centers is needed in order to compare outcomes of different surgical techniques and to establish a national protocol for optimal treatment.

## Introduction

Cleft palate (CP) is a common congenital birth defect^1^ with a prevalence of cleft lip and palate (CLP) of 6.07 per 10.000 live births and of CP of 6.78 per 10.000 live births in the Netherlands.^2^ In 2020 and 2021, respectively 217 and 209 children were born in the Netherlands with a CLP or CP.^3,4^ Children born with CP often require multiple surgeries since CP repair is essential for optimal speech development.^5–7^ The ability to separate the nasal from the oral cavity is required for the production of oral consonants, vowel sounds, and plosive, fricative and affricative phonemes.^8^ This requires complete closure of the soft palate, also known as the velum, against the posterior pharyngeal wall and is mainly achieved by retraction and elevation of the velum, resulting in a coronal closure pattern.^9^ However, movement of the pharyngeal walls also contributes to velopharyngeal closure.^9^ A circular closure pattern occurs when the lateral pharyngeal walls move toward the midline and the posterior pharyngeal wall moves anteriorly.^9^ In a sagittal closure pattern, the lateral pharyngeal wall movement is the major component of closure.^9^ In addition, some individuals have a Passavant's ridge.^9^ This is considered to be due to contraction of the inferior fibers of the superior constrictor muscle.^9^ However, in most cases, the ridge is too low to be involved in velopharyngeal closure.^9^ Research by Skolnick and Shprintzen et al. showed that the mechanism of velopharyngeal closure in patients with repaired CP and normal speech is the same as for adults with unaffected anatomy and normal speech.^10^

Approximately 20-30% of children with a history of CP repair develop velopharyngeal insufficiency (VPI).^11^ VPI is defined as inadequate closure of the velum against the posterior pharyngeal wall, resulting in hypernasality, audible nasal air emission on non-nasal consonants and reduced loudness of speech.^12^ In patients with CP, VPI is usually caused by a lack of either velar length, velar mobility or both.^12^ This can be due to the initial shortage of tissue in a CP, scar tissue, persistent non-anatomical position of the velar muscles,^12^ or after a Le Fort I osteotomy.^13^ Sometimes, VPI develops or worsens over time due to involution of the adenoid during adolescence.^9^ VPI can impact the child's ability to communicate in both social and educational settings.^11^ Children with CP have higher school absence and poorer levels of academic achievement compared to children without CP.^14^ In addition, VPI is associated with a reduced quality of life, delayed educational development, reduced earning potential, and impaired psychosocial functioning.^15^ Therefore, adequate diagnosis and treatment of VPI is of great importance.

In the Netherlands, patients with CP are treated by specialized, multidisciplinary cleft teams. These teams are based in cleft centers and include speech language pathologists (SLPs), plastic surgeons, ear-nose-throat (ENT) surgeons, maxillofacial surgeons, orthodontists, and nurse specialists. Diagnostic tools to assess VPI described in the current Dutch Cleft guideline include perceptual speech assessment (PSA) by a trained SLP to evaluate cleft speech characteristics, oral inspection, a mirror test, nasometry, nasendoscopy (NE), and videofluoroscopy (VF).^16^ During a mirror test, a small mirror is held under the nares during production of oral pressure-sensitive, non-nasal consonants.^17,18^ In case of condensation of the mirror, nasal emission is present.^17,18^ The Nasometer is a device to measure the percentage of nasalance, with results ranging from 0 to 100%.^19^ To detect hypernasality, a speech sample without nasal consonants is used.^19^ Both NE and VF are methods to directly assess velopharyngeal closure.^19^ During NE, a fiberoptic scope is used to obtain a birds’-eye view of the velopharynx during rest and real-time phonation.^19^ VF uses ionizing radiation to allow for visualization of the structure, movement, closing and timing of the velopharyngeal mechanism, usually in both a lateral, frontal and basal view.^19^ These diagnostic tests can provide valuable observations in addition to perceptual speech evaluation by a trained SLP, which is the gold standard for the assessment of cleft speech. However, these diagnostic tests lead to relatively subjective results, which could lead to heterogeneity in the diagnosis and treatment of VPI between cleft centers. In order to achieve optimal, homogeneous care of VPI in patients with CP, a national consensus on the necessary diagnostic tests, cut-off values, classification systems and optimal treatment is important. To achieve this, it is necessary to identify elements of the diagnostic and treatment workflows that require further synchronization between cleft centers, and to evaluate these matters between cleft teams. Therefore, the aim of this study was to investigate the workflow of cleft teams in the Netherlands with regard to the diagnosis and treatment of VPI in patients with CP.

## Method & Materials

The Medical Ethics Review Committee exempted this study from full review (2023.0324).

In September 2023, an online cross-sectional survey was sent to ENT surgeons, plastic surgeons and SLPs of all eight cleft centers distributed over 11 locations in the Netherlands, using Castor Electronic Data Capture (Castor EDC). A reminder was sent in October 2023. A third reminder was sent in November 2023 to two cleft centers, of which important information was still missing.

The survey questions were developed by the authors and consisted of a maximum of 25 questions. The survey questions covered the following topics: diagnostic tests used to assess VPI, use of classification systems and cut-off values to determine the most optimal treatment, treatment of VPI, and postoperative follow-up visits (Appendix A). Survey respondents were asked to indicate their specialty and the cleft center they work for in order to summarize survey responses from different specialists per cleft center.

Quantitative survey data were analyzed using SPSS (v28.0.1.1, IBM Corp, Armonk, NY, USA) and are presented as numbers (N) and proportions (%) of cleft centers. Missing data was reported. We used the “Strengthening the Reporting of Observational studies in Epidemiology” (STROBE) cross-sectional checklist when writing our report.^20^

The survey results were discussed among plastic surgeons at a special focus group meeting of the Dutch Association for Cleft Palate and Craniofacial Anomalies on November 17, 2023.

## Results

The survey was sent to a total of 47 health care professionals distributed over the eight cleft centers in the Netherlands. The health care professionals included 20 SLPs, 16 plastic surgeons, and 11 ENT surgeons. The response rate for the individual healthcare professionals was 45% (n = 21). Of these, five surveys did not contain information on the cleft center the healthcare professional worked for and were therefore excluded from data-analysis. The response rate for the cleft centers was 88% (n = 7).

**Figure 1.**
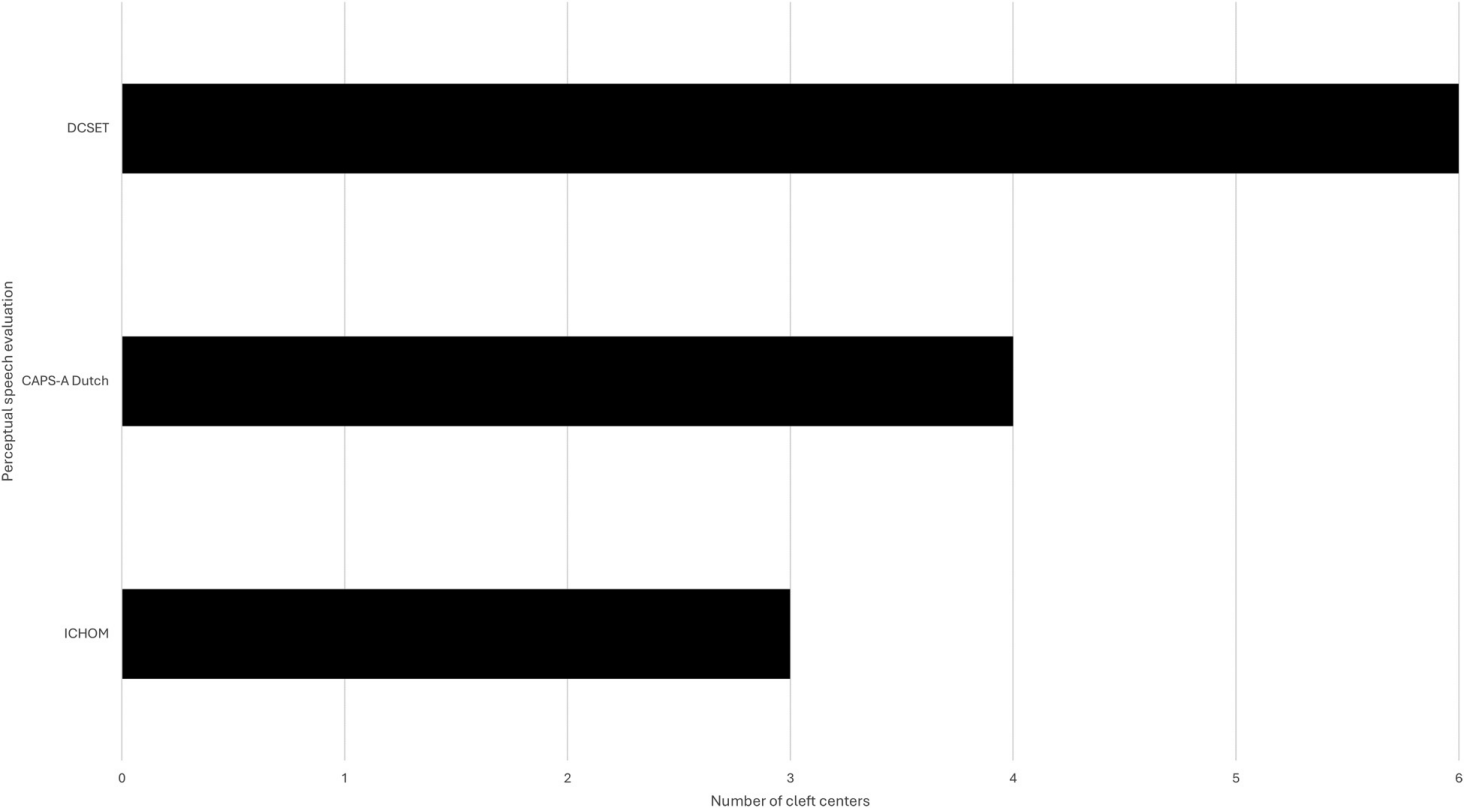
Survey results on perceptual speech evaluation regularly used for the assessment of VPI. DCSET, Dutch Cleft Speech Evaluation Test. CAPS-A Dutch, Cleft Audit Protocol for Speech-Augmented Dutch. ICHOM, percentage consonants correct (PCC) and velopharyngeal competence (VPC) from the International Consortium for Health Outcomes Measurement (ICHOM) set; VPI, velopharyngeal insufficiency.

Most cleft centers (n = 6) reported using PSA to evaluate cleft speech. As visualized in Figure 1, the Dutch Cleft Speech Evaluation Test (DCSET) was most commonly used. All cleft centers (n = 7) reported to use the mirror test and NE to assess VPI. Other frequently reported diagnostic tools include oral inspection (n = 6), patient-reported outcome measures (PROMS) (n = 5), nasometry (n = 4), and VF (n = 3). With regard to the imaging modalities, four centers reported only using NE and three centers use both NE and VF.

Six centers reported that they do not use a classification system to determine VPI severity. One center reported basing the classification of VPI on the Cleft Audit Protocol for Speech-Augmented (CAPS-A) scale for hypernasality, which ranges from zero to four.

**Figure 2.**
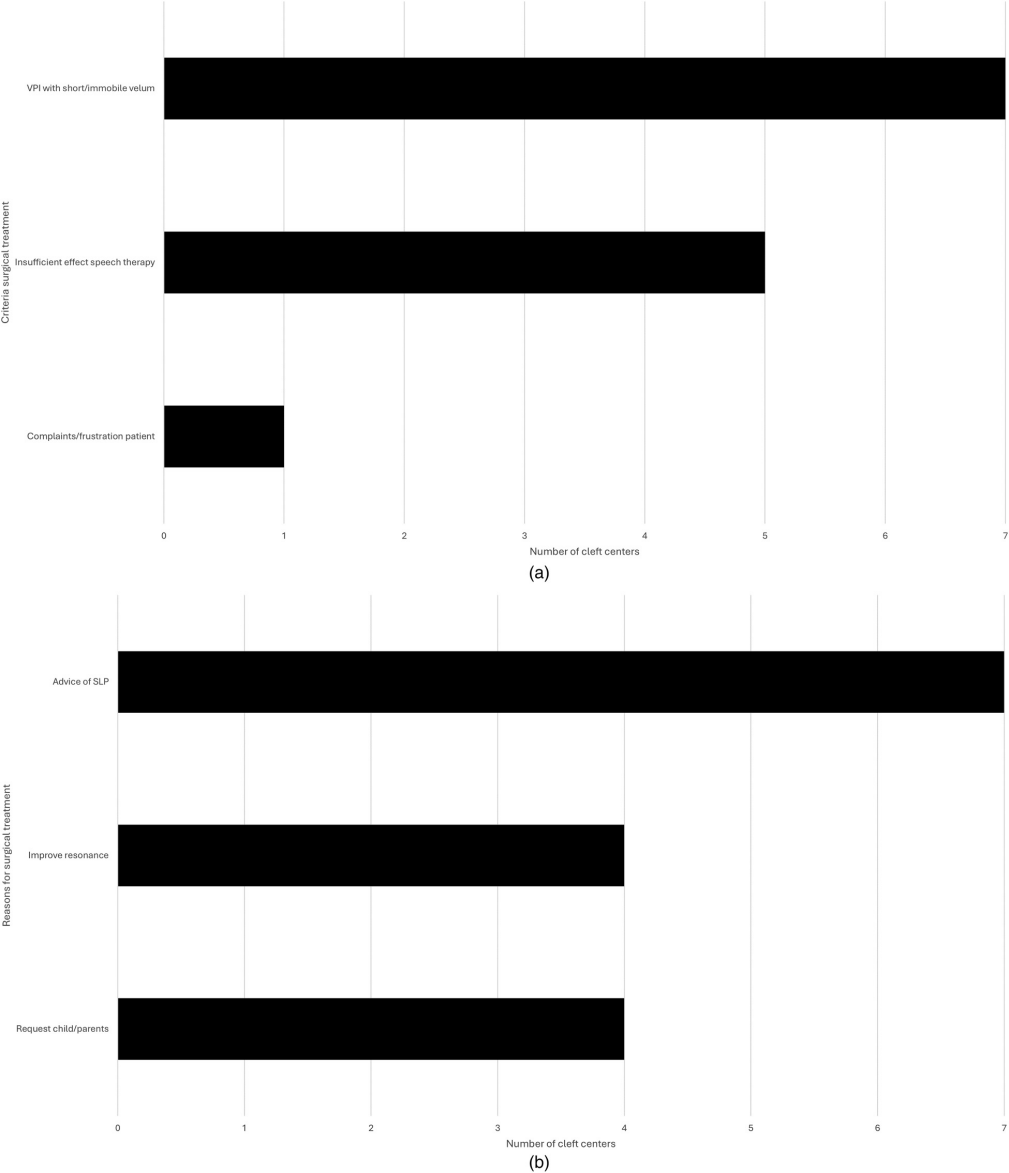
Criteria and reasons to choose for surgical treatment of VPI. VPI, velopharyngeal insufficiency.

Most centers (n = 6) reported that they did not use predetermined cut-off values of the diagnostic tests to determine whether or not a patient required surgical treatment for the VPI. However, several criteria are reportedly used by the cleft centers to determine whether a patient requires surgical treatment. These criteria are visualized in Figure 2a. Shared decision making was also reported to be an important factor in this process (n = 2). Reported reasons for the eventual decision for surgical treatment are presented in Figure 2b.

In almost all centers (n = 6), children receive speech therapy prior to surgical treatment. The duration of speech therapy before surgery varies between cleft centers, ranging from 3 months to 2 years.

**Figure 3.**
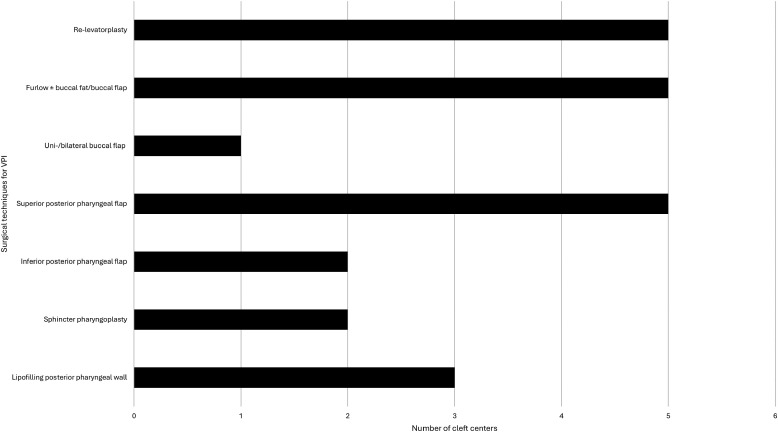
Survey results for VPI treatment. VPI, velopharyngeal insufficiency.

Many different surgical techniques for the treatment of VPI were reported. The most frequently used techniques include the Furlow palatoplasty with or without a buccal flap and/or buccal fat (n = 5), intravelar veloplasty (re-levatorplasty) (n = 5), and superior posterior pharyngeal flap (n = 5), followed by lipofilling of the posterior pharyngeal wall (n = 3). Most centers (n = 5) reported to use palatoplasty as well as pharyngoplasty techniques. One center reported to use only palatoplasty techniques and for one center information regarding the surgical techniques was missing. The chosen surgical technique was reported to depend on the underlying anomaly, velar anatomy and function, and previous surgical treatment. Figure 3 provides an overview of the survey results for VPI treatment.

During postoperative follow-up visits, patients are often seen by the surgeon (either a plastic surgeon or maxillofacial surgeon) and sometimes by an SLP or an ENT surgeon. The first postoperative follow-up was reported to usually take place between 1 and 6 weeks after surgery. This follow-up visit includes an oral inspection in all seven centers. Other tests reportedly used during this visit are PROMs, mirror test, nasometry, and PSA using the CAPS-A Dutch. The second postoperative visit usually takes place between 2 weeks and 6 months. With regard to a third postoperative visit, one center reported scheduling this only when necessary and one center does not schedule patients for a third visit. In four centers, the third follow-up visit takes place between one month and 1 year after surgery.

## Discussion

This survey-based study examined the workflow of cleft teams in the Netherlands regarding the diagnosis and treatment of VPI in patients with CP. Significant discrepancies were found between cleft centers. Most centers reported that they did not use predetermined cut-off values to determine whether a patient required surgical treatment for VPI. There was considerable variation in the duration of speech therapy before surgery, ranging from 3 months to 2 years. Additionally, many centers used a wide range of different surgical techniques for VPI treatment. Postoperative follow-up procedures also varied greatly between centers, making it more challenging to evaluate surgical outcomes and assess the impact on patients, families, and SLPs.

The results of this survey study reflect the current practice of Dutch cleft teams regarding diagnosis and treatment of VPI in patients with CP and highlight the matters on which the national consensus could be increased. A national consensus based on the currently available literature and guidelines, supplemented with expert opinion on the subjects for which the literature lacks a clear recommendation, can lead to optimal, nationally homogeneous care of VPI in patients with CP. In order to achieve this, consensus meetings among professionals are needed.

The results indicate that Dutch cleft centers most commonly use PSA, NE, oral inspection, nasometry, and the mirror test to assess VPI in patients with CP. These findings align with the Dutch Cleft guideline, which recommends using all of these diagnostic tools to assess VPI.^16^ In the recent literature, PSA by a trained SLP^15,21–34^ and NE^15,22–27,29,30,32–34^ are frequently described for the assessment of VPI in patients with CP. Interestingly, nasometry^15,26,27^ and the use of a mirror test^28^ are not often mentioned in the literature. This may be caused by these tests being more valuable for follow-up during treatment, rather than as a diagnostic tool, or because they are considered part of the PSA and therefore not reported separately. In addition, many centers do not have access to a Nasometric device. In the survey, only three centers reported regular use of VF. This aligns with the guideline's recommendation to use VF only when NE fails or in case additional information on velar function is needed.^16^ Despite its ability to provide valuable insights into velopharyngeal function and its potential for better patient acceptance compared to NE, the use of VF is rarely described in the literature as a standard tool to evaluate VPI.^25,29,31,33^ This is likely due to concerns about radiation exposure. One center did not report to use PSA for the evaluation of VPI. Since the survey was not filled in by one of the SLPs of this cleft team, there is a possibility that this result reflects missing data, rather than PSA not being used as a diagnostic tool in this specific center.

Although not specifically recommended by the Dutch Cleft guideline,^16^ the use of the CAPS-A Dutch speech test was frequently reported by the cleft centers. The CAPS-A Dutch was developed only recently, after publication of the latest version of the Dutch Cleft guideline in 2012.^16^ The advantage of the CAPS-A is that it uses scalar values to assess multiple speech parameters associated with velopharyngeal function, such as hypernasality, nasal air emission, and compensatory misarticulations.^35^ Furthermore, systematic training in the use of the CAPS-A in American, and Canadian or British English results in acceptable levels of reliability for the parameters most relevant to the assessment of velopharyngeal function.^35^ In the Netherlands, SLPs attend consensus training twice a year for the assessment of cleft speech using the DCSET, percentage consonants correct, and velopharyngeal competence from the International Consortium for Health Outcomes Measurement (ICHOM) set and CAPS-A Dutch, the latter of which will soon be validated. Therefore, we suggest the incorporation of the CAPS-A Dutch in the Dutch Cleft guideline recommendations as soon as it has been validated.

Similarly, PROMs were reported by most cleft centers as part of the diagnostic process, despite not being included in the Dutch Cleft guideline. The inclusion of PROMs is essential for providing high-quality, patient-centered care, and can improve health-related quality of life.^36^ By capturing outcomes directly relevant to patients, PROMs offer insights that may be more meaningful than clinical measures alone.^36^ We therefore recommend incorporating PROMs into the Dutch Cleft guideline to enhance patient-centred assessment and care.

Several classification systems for VPI severity have been described in the literature. Nevertheless, only one cleft center reported using a classification system for VPI severity based on the CAPS-A scale for hypernasality. Classification systems described in the literature include the Pittsburgh Weighted Speech Scale,^21,25^ the PSA scale,^33^ and 5-point,^15,22,27^ 4-point,^15,23^ 3-point^15,31^ or dichotomous^15^ numerical or Likert-type rating scales based on nasal air flow and resonance.^15^ The fact that so many scales have been described in the literature, however, indicates that there is no ideal option to score speech yet.

None of the cleft centers reported using predetermined cut-off values based on the diagnostic test results to decide whether patients receive speech therapy or surgical therapy. This may be due to the relatively subjective nature of the test results and the lack of clear recommendations from both the Dutch Cleft guideline^16^ and the literature. Nevertheless, all centers showed relative agreement on the factors that play a role in the decision making to proceed with surgical treatment for the VPI. Furthermore, the evaluation of cleft speech characteristics using the DCSET and CAPS-A Dutch can be used to determine the need for speech therapy. In addition to scoring speech, velopharyngeal gap size could be assessed on imaging, such as NE,^22^ VF^31^ or magnetic resonance imaging (MRI)^37^ in order to further objectify the clinical assessment of VPI. The use of a classification system, together with predetermined, objectively measured cut-off values, such as nasometry values, CAPS-A scores or objective imaging measurements, could both improve the consensus on the treatment planning and allow for comparison of pre- and post-treatment outcomes between cleft centers. It is important to note that Nasometric measurements will only result in homogeneous cut-off values of meaning in case all cleft centers use the same Nasometric device and subtests. Of the imaging modalities, only VF and NE are reportedly used by the cleft centers and recommended by the Dutch Cleft guideline.^16^ However, unsedated velopharyngeal MRI has been suggested in the literature for direct assessment of both velopharyngeal gap size, velar mobility and the position of the velar muscles.^37^ This diagnostic tool has been described to be successfully applicable to patients from the age of four.^38–41^ Future research should focus on the applicability of this reproducible and radiation free diagnostic tool^37^ for the assessment of VPI in patients with CP. Prior to national implementation of classification systems and measurements on imaging in the diagnostic process, consensus on which scales to use, which measurements to do and training of professionals in the use of the chosen scales and imaging assessment techniques is of importance.

It is essential to consider that socially unacceptable speech characteristics, particularly those related to resonance in VPI, are the primary factors guiding treatment decisions. Therefore, PSA by a trained SLP and patient-reported outcomes (PROs) play a crucial role in VPI assessment. Additional diagnostic tools, along with the possible use of a classification system and specific criteria for surgical intervention, can further support clinical evaluation and treatment planning.

Regarding VPI treatment, almost all cleft centers reported that their patients receive speech therapy prior to surgery, which is in line with the recommendation of the Dutch Cleft guideline.^16^ Reported duration of speech therapy varied between 3 months and 2 years, whereas the minimum duration recommended by the Dutch Cleft guideline is six months.^16^ The heterogeneous survey responses could be explained by the fact that both the duration and the success of speech therapy may depend on patient-specific and circumstantial factors. Patients with VPI that is mainly structural in nature may require less or no speech therapy prior to surgical treatment compared to patients with VPI that is more functional in nature. Information on the level of speech development, understanding of patients regarding instructions, or their motivation to attend speech therapy was not taken into account in this survey study. Furthermore, hearing was also not taken into consideration. Impaired hearing abilities can have an impact on speech and language development,^42^ and otitis media can be prevalent in the CP population.^43^ These matters could have had an impact on the use of speech therapy by the cleft centers. Nevertheless, increased standardization of the treatment workflow between the cleft centers could lead to more homogeneous use of speech therapy.

Several surgical techniques have been reported for the treatment of VPI in patients with CP. All centers reported the use of palatoplasty as well as pharyngoplasty techniques, except for one center that only uses palatoplasty techniques. Information regarding surgical techniques was missing from one center. Common surgical techniques described in the literature for the treatment of VPI in patients with CP include pharyngeal flap, sphincter pharyngoplasty, palatoplasty, and posterior pharyngeal wall augmentation.^44^ A systematic review by De Blacam et al^44^ found no significant differences in speech outcomes or complications, including obstructive sleep apnea (OSA), among these procedures. Similarly, a meta-analysis by Collins et al^45^ showed no clear advantage in speech outcomes or OSA risk between sphincter pharyngoplasty and pharyngeal flap. Repositioning the velar muscles, combined with palate lengthening, has been shown to effectively improve velopharyngeal function.^44^ A postoperative situation in which both the anatomical and functional characteristics of the velum are as close to a non-cleft situation is likely to be most favorable. Since the literature does not show a clear advantage of one surgical technique over the other, or an advantage of a combination of techniques over a single technique, the Dutch Cleft guideline recommends an intravelar veloplasty as the first choice of surgical treatment for VPI after primary CP repair.^16^ Only in case of persistent VPI despite previous redo-palatoplasty, pharyngoplasty is recommended as the next step.^16^ In case of pharyngeal flap, the nasopharyngeal port is obstructed and velopharyngeal closure is achieved through medial contraction of the lateral pharyngeal walls on either side of the flap.^44^ Therefore, this technique may be more suitable for neuromuscular causes of VPI or patients with severe hypotonia, for example patients with 22q11.2 deletion syndrome, in which there is minimal velar mobility and no positive result of velar retroposition is to be expected. In addition to these surgical techniques, lipofilling is reportedly used by three centers. Lipofilling has been described in the literature as minimally invasive treatment option for mild to moderate VPI.^46^ However, homogenous evaluation of complications, outcomes, and long-term efficacy is limited.^46^ Therefore, lipofilling is recommended by the Dutch Cleft guideline only to be used in case there are no other surgical options.^16^ The literature has not yet resulted in a general consensus regarding choice of optimal surgical treatment for VPI and the recommendations of the Dutch Cleft guideline therefore remain inconclusive. This may explain the heterogeneity of survey responses regarding surgical techniques used to treat VPI. Survey responses indicate that the chosen surgical technique depends on the underlying anomaly, velar anatomy and function, and previous surgical treatment. However, it is unclear how the underlying anomaly and velopharyngeal function were assessed. Future research should focus on identifying the most effective treatment for each patient and further investigating optimal surgical techniques for VPI management in patients with CP.

With regard to the postoperative follow-up, survey responses showed a consensus on the multidisciplinary approach, which is in line with the Dutch Cleft guideline.^16^ Oral inspection, mirror tests, PROMs, and PSA (various tests) were reported by most centers in the survey to be used both before and after surgical treatment. Furthermore, NE and VF were not reported by the cleft teams to be used for postoperative assessment of VPI. This is in line with the recommendation of the Dutch Cleft guideline to repeat all diagnostic tests used prior to surgery, except for NE and/or VF, 1 year after surgical treatment.^16^ The reported timing of postoperative follow-up visits was heterogeneous. This could be partly due to postoperative visits that coincide with visits at certain ages following local protocol, which leads to the postoperative visits not being registered as such. A more standardized process for the assessment of VPI may also allow for more homogeneity in postoperative follow-up protocols among cleft centers. However, it is important to keep room for individualized approaches in the postoperative follow-up.

The aim of our study was to obtain complete survey responses for each cleft center by combining survey responses from individual health care professionals. Regarding the individual health care professionals, our response rate of 45% is in line with the average response rate of 46% ± 25% for web-based surveys reported in the systematic review by Meyer et al. ^47^ Some questions were specific to either surgeons or SLPs, which may have reduced the motivation of individual healthcare professionals to complete the entire survey. In addition, cleft teams consist of more than one plastic surgeon and also more than one SLP and/or ENT surgeon. Therefore, it is likely that per specific specialty, only one of the colleagues of each cleft team responded to the survey. We achieved a response rate for the cleft centers of 88% and the summarized survey responses of individual healthcare professionals provide extensive information on VPI diagnosis and treatment for seven out of eight cleft centers in the Netherlands.

A limitation of our study is the small geographical area where our study was conducted, which confines the generalizability of the findings. Another limitation is that the reliability, experience and training of individual SLPs is unknown. However, in the Netherlands, the specialized SLPs that are connected to cleft teams attend consensus training twice a year for the assessment of cleft speech using the DCSET, ICHOM, and CAPS-A Dutch. Furthermore, responses of individual health care professionals from the same cleft team sometimes differed, which made summarizing the results per cleft center challenging for a few topics. Nevertheless, our study evaluates the process of diagnosis and treatment of VPI in patients with CP in the Netherlands and highlights the elements on which the national consensus may be increased.

## Conclusion

Further standardization of the diagnostic process and treatment planning of VPI in patients with CP between Dutch cleft centers is needed. This will allow establishment of a national protocol for optimal treatment in all centers, comparison of outcomes of different surgical techniques and objective evaluation of outcomes both within cleft centers and between teams.

## Supplemental Material

sj-docx-1-cpc-10.1177_10556656251341757 - Supplemental material for Dutch Workflow for Diagnosis and Treatment of Velopharyngeal Insufficiency in Patients with Cleft Palate—A Survey Study Supplemental material, sj-docx-1-cpc-10.1177_10556656251341757 for Dutch Workflow for Diagnosis and Treatment of Velopharyngeal Insufficiency in Patients with Cleft Palate—A Survey Study by JJ Peters, SA van der Kooij, CDL van Gogh, MJ Coerts, JPW Don Griot, CM Moues-Vink, T Wagner, RM Schols, B van Nimmen, MM Pleumeekers, M Ruettermann, EC Paes, TR De Jong and CC Breugem in The Cleft Palate Craniofacial Journal

sj-docx-2-cpc-10.1177_10556656251341757 - Supplemental material for Dutch Workflow for Diagnosis and Treatment of Velopharyngeal Insufficiency in Patients with Cleft Palate—A Survey Study Supplemental material, sj-docx-2-cpc-10.1177_10556656251341757 for Dutch Workflow for Diagnosis and Treatment of Velopharyngeal Insufficiency in Patients with Cleft Palate—A Survey Study by JJ Peters, SA van der Kooij, CDL van Gogh, MJ Coerts, JPW Don Griot, CM Moues-Vink, T Wagner, RM Schols, B van Nimmen, MM Pleumeekers, M Ruettermann, EC Paes, TR De Jong and CC Breugem in The Cleft Palate Craniofacial Journal
