# Supplementary material for: Dutch Workflow for Diagnosis and Treatment of Velopharyngeal Insufficiency in Patients with Cleft Palate—A Survey Study
Source: Cleft Palate Craniofac J. 2025 May 19;63(6):1553–61. doi: 10.1177/10556656251341757 (PMC13176485; doi:10.1177/10556656251341757)
Supplement: sj-docx-1-cpc-10.1177_10556656251341757 - Supplemental material for Dutch Workflow for Diagnosis and Treatment of Velopharyngeal Insufficiency in Patients with Cleft Palate—A Survey Study [file sj-docx-1-cpc-10.1177_10556656251341757.docx]

# Appendix A – Survey

**Introduction**

By filling in the survey you accept the following terms:

- I have read the information letter. I was able to ask questions. My questions have been sufficiently answered. I had enough time to decide whether or not to participate.
- I am aware that filling in the survey is voluntary. I am aware that I can decide at any moment not to participate anymore. I do not have to give an explanation.
- I give permission to collect and to use my data for the purposes stated in the information letter.
- I give permission to store my data for the duration of 10 years at the XXXXXX location XXXX.
- I wish to participate in this research.

**Question 1**

I give permission to be approached in the future for other research.

- Yes
- No

**Question 2**

In which hospital do you work?

**Question 3**

What is your specialty?

- Speech Language Pathologist
- Plastic surgeon
- Maxillofacial surgeon
- ENT surgeon
- Other

**Question 4**

At what age is the soft palate usually repaired at your center?

- 6-9 months
- 9-12 months
- Other
- I do not know

**Question 5**

In case of a cleft lip and palate, is a vomerine flap used during primary lip repair?

- Yes
- No
- Sometimes
- I do not know

**Question 6**

Is the entire palate repaired before the age of 12 months?

- Yes
- No (the hard palate is left open until a later age)
- Other
- I do not know

**Question 7**

Which surgical techniques are used in your center for primary cleft palate repair?

- Furlow
- Von Langenbeck
- Other
- I do not know

**Question 8**

Which speech tests are used for the assessment of VPI?

- CAPS-A Dutch
- ICHOM
- DCSET
- Other
- I do not know

**Question 9**

Which other diagnostic tests are used for the assessment of VPI?

- Mirror test
- Nasometry
- Oral inspection
- Nasendoscopy
- Videofluoroscopy
- PROMs
- Other
- I do not know

**Question 10**

Does your cleft team use a classification system to determine the severity of the VPI?

- Yes
  - Which classification system is used?
- No

**Question 11**

Do patients always have speech therapy before surgical therapy for the VPI?

- Yes
  - For how long do patients usually have speech therapy prior to surgical therapy?
- No

**Question 12**

What criteria does your team use to decide whether or not a patient requires surgical treatment for the VPI?

**Question 13**

Does your team use any predetermined cut-off values based on the diagnostic tests to make this decision?

**Question 14**

What are the most important reasons to choose for surgical treatment of the VPI?

- Improve resonance
- Request SLP
- Request patient or parent(s)/caregiver(s)
- Other

**Question 15**

Based on which factors do you determine the surgical technique that will be used for treating the VPI?

**Question 16**

Which surgical techniques for the treatment of VPI are used in your cleft center?

- Re-levatorplasty
- Furlow palatoplasty
- Furlow palatoplasty + buccal flap
- Furlow palatoplasty + buccal flap + buccal fat
- Superior posterior pharyngeal flap
- Inferior posterior pharyngeal flap
- Sphincter pharyngoplasty
- Lipofilling posterior pharyngeal wall
- Other
- I do not know

**Question 17**

At what moment does the first postoperative follow-up visit take place?

- 1 week postoperative
- 3 weeks postoperative
- 6 weeks postoperative
- Other

**Question 18**

At what moment does the second postoperative follow-up visit take place?

- 3 months postoperative
- 6 months postoperative
- 9 months postoperative
- Other

**Question 19**

At what moment does the third postoperative follow-up visit take place?

- 1 year postoperative
- No third follow-up visit
- Other

**Question 20**

Which specialists are involved during the first postoperative follow-up visit?

- SLP
- Plastic surgeon
- Maxillofacial surgeon
- ENT surgeon
- Other

**Question 21**

Which specialists are involved during the second postoperative follow-up visit?

- SLP
- Plastic surgeon
- Maxillofacial surgeon
- ENT surgeon
- Other

**Question 22**

Which specialists are involved during the third postoperative follow-up visit?

- SLP
- Plastic surgeon
- Maxillofacial surgeon
- ENT surgeon
- Other

**Question 23**

Which diagnostic tools are used for the assessment of VPI during the first postoperative follow-up visit?

- CAPS-A Dutch
- ICHOM
- DCSET
- Mirror test
- Nasometry
- Oral inspection
- Nasendoscopy
- Videofluoroscopy
- PROMs
- Other

**Question 24**

Which diagnostic tools are used for the assessment of VPI during the second postoperative follow-up visit?

- CAPS-A Dutch
- ICHOM
- DCSET
- Mirror test
- Nasometry
- Oral inspection
- Nasendoscopy
- Videofluoroscopy
- PROMs
- Other

**Question 24**

Which diagnostic tools are used for the assessment of VPI during the third postoperative follow-up visit?

- CAPS-A Dutch
- ICHOM
- DCSET
- Mirror test
- Nasometry
- Oral inspection
- Nasendoscopy
- Videofluoroscopy
- PROMs
- Other

**Question 25**

Do you ever undo previous surgical interventions (e.g. cleave a pharyngeal flap)?

- Yes
  - What are the most common reasons to do so?
  - What is usually the effect on the VPI?
- No

**Outro**

Thank you for filling in the survey.
